# Transparent Blend of Poly(Methylmethacrylate)/Cellulose Acetate Butyrate for the Protection from Ultraviolet

**DOI:** 10.3390/polym8040128

**Published:** 2016-04-14

**Authors:** Raouf Mahmood Raouf, Zaidan Abdul Wahab, Nor Azowa Ibrahim, Zainal Abidin Talib, Buong Woei Chieng

**Affiliations:** 1 Department Of Physics, Faculty of Science, Universiti Putra Malaysia, 43400 UPM Serdang, Selangor, Malaysia; zaidan@upm.edu.my (Z.A.W.); zainalat@upm.edu.my (Z.A.T.); 2 Materials Engineering Department, College Of Engineering, Al-Mustansiriyah University, 10047 Bab Al Muadham, Baghdad, Iraq; 3 Department Of Chemistry, Faculty of Science, Universiti Putra Malaysia, 43400 UPM Serdang, Selangor, Malaysia; norazowa@upm.edu.my (N.A.I.), chieng891@gmail.com (B.W.C.); 4 Materials Processing and Technology Laboratory, Institute of Advanced Technology, Universiti Putra Malaysia, 43400 UPM Serdang, Selangor, Malaysia

**Keywords:** absorbance, amorphous, optical properties, thermal properties, transmittance

## Abstract

The use of transparent polymers as an alternative to glass has become widespread. However, the direct exposure of these materials to climatic conditions of sunlight and heat decrease the lifetime cost of these products. The aim of this study was to minimize the harm caused by ultraviolet (UV) radiation exposure to transparent poly(methylmethacrylate) (PMMA), which usually leads to changes in the physical and chemical properties of these materials and reduced performance. This was achieved using environmentally friendly cellulose acetate butyrate (CAB). The optical, morphological, and thermal properties of CAB blended with transparent PMMA was studied using UV-VIS spectrophotometry, scanning electron microscopy, X-ray diffraction, dynamic mechanical analysis, and thermal gravimetric analysis. The results show that CAB was able to reduce the effects of UV radiation by making PMMA more transparent to UV light, thereby preventing the negative effects of trapped radiation within the compositional structure, while maintaining the amorphous structure of the blend. The results also show that CAB blended with PMMA led to some properties commensurate with the requirements of research in terms of a slight increase in the value of the modulus and the glass transition temperature for the PMMA/CAB blend.

## 1. Introduction

Polymer blends are able to provide materials with extensive functional properties beyond the range that can be gained from single polymer equivalents. Research has confirmed that di-block copolymers are more efficient in decreasing the phase size and tri-block copolymers are more effective in developing the mechanical properties [1]. They are also important from the environmental and economic points of view [2,3,4]. Currently, researchers tend to reduce the effect of ultraviolet rays on polymers by mixing them with substances that have good ultraviolet stability [5,6,7]. Cellulose acetate butyrate (CAB) is consider to be one of the most plentiful natural renewable resources with high ultraviolet stability, very high impact strength, low moisture absorption, and a relatively low glass transition temperature in comparison with other cellulose esters; therefore, it has attracted attention as a key to the problems experienced by some polymers [8]. CAB has been used as a plasticizer in some inorganic studies to decrease cracks, corrosion, the effects of ultraviolet radiation, and the effects of atmospheric oxygen. As well as being a wood filler emulsion [9,10], it is also used to prepare light-sensitive printing paper; this works by exposing the paper to an ultraviolet source to enhance dye adhesion, which acts as a binder between the lacquer and the metal surface [11,12]. CAB blends show exceptional transparency with a large number of transparent polymers. Furthermore, CAB’s partial miscibility and good interface-forming ability are responsible for significant increases in both mechanical and thermal properties. This has been attributed to H-bonding via the hydroxyl groups of CAB which have specific interactions with effective mixture groups [2].

Polymers are vulnerable to ultraviolet radiation degradation because many types of bonds in organic polymers are able to absorb ultraviolet light [13]. Polymethylmethacrylate (PMMA) is a transparent polymer possessing many excellent physical properties; in particular, it has low density and high light transmittance. PMMA is an artificial acrylic resin formed from the polymerization of methyl-methacrylate and has high optical clarity. In external applications, PMMA exposed to climatic conditions of solar ultraviolet radiation and hot weather undergoes changes in its external appearance and functionality, such as fogging and cracks due to chain scission in the polymer structure [14]. The scission depends on the amount of energy that absorbed by PMMA [15]. At a low irradiation dose, a crosslinking reaction occurs between the ester side groups in PMMA polymer molecules. With a moderate irradiation dose, side-chain degradation from the main polymer chain occurs, yielding mechanical densification of the polymeric material due to van der Waals forces, with a subsequent increase in some optical properties. At a high irradiation dose, polymer main chain scission occurs, followed by total defragmentation of the polymer structure [16,17]. During irradiation, the ultraviolet absorption of PMMA increases within a band around 285 nm, due to carbonyl groups [15]. The goal of this work is looking for the best blending ratio of PMMA/CAB in order to make the blend more transparent to UV radiation (less absorbance) by taking advantage of the unusual optical properties of CAB.

## 2. Materials and Methods

White powder cellulose acetate butyrate (CAB; average *M*_n_ ~ 12,000) and white powder poly (methylmethacrylate) (PMMA; average *M*_w_ ~ 120,000 by GPC) were supplied by Sigma-Aldrich (Saint Louis, MO, USA).

The CAB/PMMA blend was prepared using a twin screw Thermo-Haake Poly Drive (karlsruhe, Germany) (*D* =19.05 mm) and a hot press from Hsin-Chi Machinery Co. Ltd. (Dong Guan, Taiwan). The materials were dried in a vacuum oven at 50 °C for 4 h before mixing. To prepare PMMA/CAB samples, a fixed weight of PMMA was melt-kneaded in the extruder at a rotation rate of 50 rpm at 130 °C for 10 min. Then, variable percentage weights of CAB (5%, 7%, 9%, *etc.*, to 25%) were added to the molten PMMA. Mixing continued until a constant torque was reached, which took about 10–15 min. The samples were transparent and homogeneous. After that, each blended sample was pressed in a hot press at 110 KPa and 130 °C to form a sheet 70 mm × 90 mm, 1 mm thick.

The nature of the transparency and absorbance of the samples was ascertained using ultraviolet-visible (UV-VIS) spectroscopy (Shimadzu UV-3600 spectrophotometer, Tokyo, Japan) according to ASTM D1003. Surface topography images were obtained by scanning electron microscopy (SEM) studies on a Hitachi S-3400N (Makuhari Messe, Japan) microscope. Sample morphology and crystallinity were determined by XRD analysis using a X-ray diffractometer (Philips/X’Pert Pro Panalytical-PW 3040/60 MPD, Almelo, Netherlands). The diffractometer data were obtained from 2Ɵ = 20° to 80° at a scanning speed of 5°/min. Thermogravimetric analysis (TGA) was undertaken on a TGA/DSC1 STAR System (Columbus, OH, USA) at a heating rate of 20 °C/min from room temperature to 1000 °C in a continuous highly-pure nitrogen atmosphere. Dynamic mechanical analysis (DMA) was performed on a PerkinElmer Pyris Diamond (Waltham, MA, USA) apparatus in tension mode at a frequency of 1 Hz and a heating rate of 5 °C/min in a liquid nitrogen atmosphere.

Full scans over the ultraviolet and visible spectra were made from 220 to 800 nm for highly-transparent pure PMMA samples using a Shimadzu UV-3600 spectrophotometer. The results show that the absorbance peak in the ultraviolet region for pure PMMA was at 226 nm, which represents the UV damage threshold for the polymer [18,19,20], whilst the transmittance peak in the visible region for pure PMMA was 798 nm. The transmittance peak indicates the actual performance for pure PMMA [19]. The absorbance and transmittance peak values were adopted for all subsequent measurements on the blended samples under study, in addition to the transparency.

## 3. Results and Discussion

### 3.1. UV-VIS Spectroscopy

**Figure 1:**
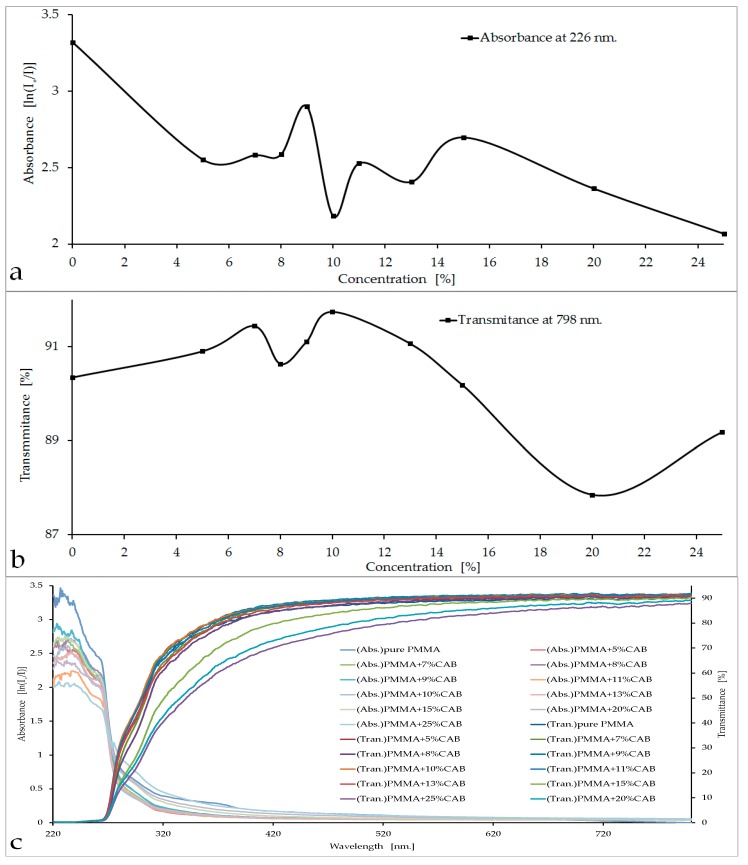

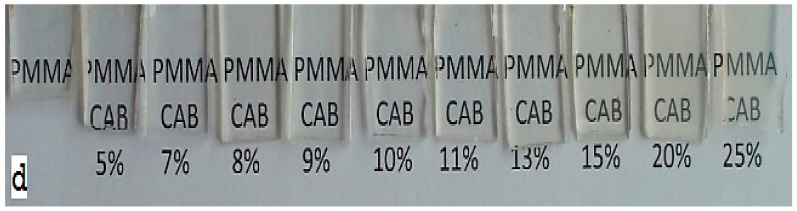
(**a**) Absorbance of PMMA and CAB in different concentrations at 226 nm; (**b**) transmittance of PMMA and CAB in different concentrations at 798 nm; (**c**) PMMA/CAB absorbance and transmittance spectrum with concentration; and (**d**) PMMA/CAB sheets in different concentrations.

The absorbance and transmittance curves, as well as the spectra and the visual appearance of the PMMA/CAB blends, are shown in Figure 1.

Initially, the CAB concentrations in PMMA were 5%, 10%, 15%, 20%, and 25%. The curve of absorbance *vs.* concentration for these five ascending concentrations showed that the lowest value of absorbency was at 10% CAB, *i.e.* about 2.184 (knowing that the absorption of pure PMMA is 3.321), with an increase in transparence up to 92%. The next step was to prepare new samples (7%, 8%, 9%, 11%, 13%, and 15%) to make a full survey of the low-absorbance area (10% CAB). Three samples were tested from each concentration and then measurements were taken to calculate the values of absorbance and transmittance. All absorbance and transmittance results were identical to those previously reported in Figure 1a,b. The absorbance curve at 226 nm showed a clear decrease with concentration, especially at 10% and 25% (Figure 1a). Conversely, fluctuations in the value of optical transmittance occurred in the visible region, as is evident in Figure 1b with two clear peaks at 7% CAB and 10% CAB. The sharp decline in transmittance curve happened at 25% CAB because the blend was heterogeneous at high concentrations [21]. The selection of PMMA/10%CAB as the best blend concentration meets the required purpose of the work, which is achieve the lowest absorbency of optical radiation within the UV region, especially at the damage threshold of PMMA (226 nm). Decreasing the absorbance value in the damage threshold area reduced the effect of trapped harmful rays inside the material and made the material more transparent to these rays, meeting the purpose of research [22,23]. Conversely, there was a slight increase in the value of transparency in the visible region, which should improve PMMA performance.

In the PMMA/CAB blends, it was noted that the samples were transparent up to 20% CAB; after that, the samples started to become hazy with increasing CAB concentrations in PMMA. Samples with concentrations greater than 20% were discarded because they did not meet the required purpose (Figure 1d).

### 3.2. Scanning Electron Microscopy

**Figure 2:**
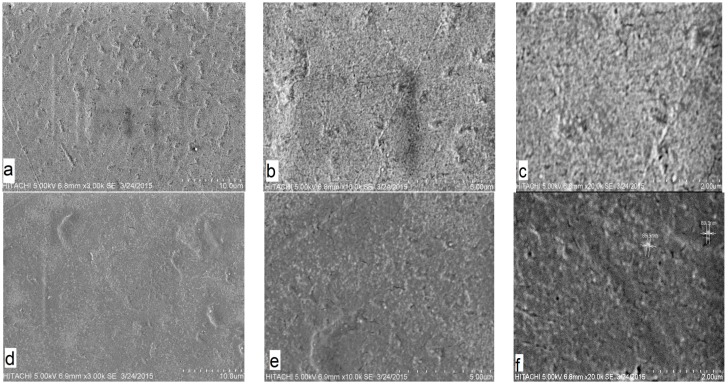
SEM images for PMMA and PMMA/10%CAB blend with different scale bars: (**a**) PMMA/10 µm; (**b**) PMMA/5 µm; (**c**) PMMA/2 µm; (**d**) PMMA/10%CAB/10 µm; (**e**) PMMA/10%CAB/5 µm and (**f**) PMMA/10%CAB/2 µm.

The SEM images taken for the internal morphological study of pure PMMA and PMMA/10%CAB blend are shown in (Figure 2). The SEM images for each sample are shown at three different scale bar (2, 5, 10 µm). As can be seen in the pure PMMA images, there were uniform morphological features, indicating a single material (Figure 2a–c) [24].

The single phase of the PMMA/10%CAB blend surface with inconsistent dispersal of CAB in PMMA domains appear as white areas, without clear phase separation, which indicated average miscibility of CAB with PMMA on a microscopic scale. Macro phase separation in the PMMA/CAB blends did not occur at the mixing and injection molding temperature (130 °C) [25]. Figure 2d–f show the inconsistent dispersal of CAB within PMMA domains with the presence of CAB conglomerates, but these conglomerates or clusters were no larger than 89.9 nm, as shown in Figure 2f; therefore, CAB dispersion in PMMA showed intermediate continuity, which could be attributed to the formation of hydrogen bonds between the carbonyl groups of PMMA and the free hydroxyl groups of CAB [26,27,28].

### 3.3. X-Ray Diffraction

**Figure 3:**
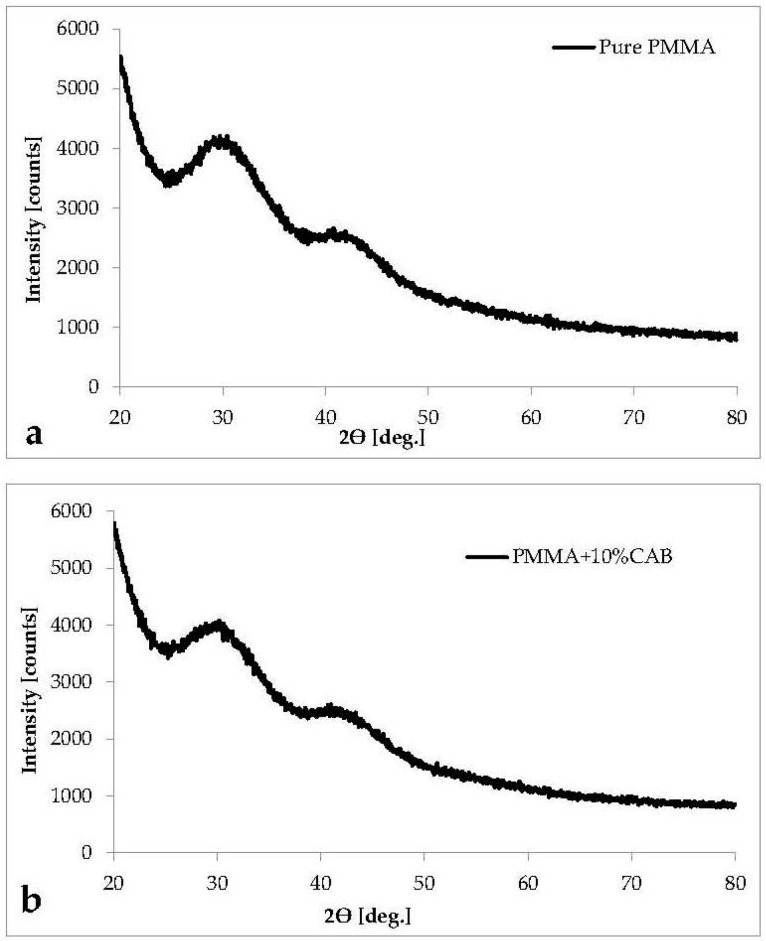
XRD pattern for: (**a**) pure PMMA, and (**b**) PMMA/10%CAB.

The X-ray diffraction pattern of pure PMMA and PMMA/10%CAB blend are shown in (Figure 3). The pattern in (Figure 3a) shows that there are no sharp peaks. The broadened background scattering area of pure PMMA indicates its amorphous nature and non-crystalline structure [29,30]. So, the lack of change observed from the pure polymer to the blend indicates that CAB did not change the basic random compositional structure of the original polymer because of CAB amorphous structure (Figure 3b). This corresponds with previous data indicating that the spherical clusters were no larger than 89.9 nm in PMMA/10%CAB are not crystalline regions.

### 3.4. Dynamic Mechanical Analysis

The dynamic mechanical analysis involves studying the dynamic mechanical reaction of viscoelastic materials to sinusoidal changeable strain and is useful in improving materials [31]. The dynamic modulus indicates the intrinsic stiffness of the material under dynamic loading conditions. Some polymeric blends are well-suited to this test due to their monosyllabic identical phase. Conversely, most polymer blends form two phases because of incompatibility between the blend elements [32]. 

**Figure 4:**
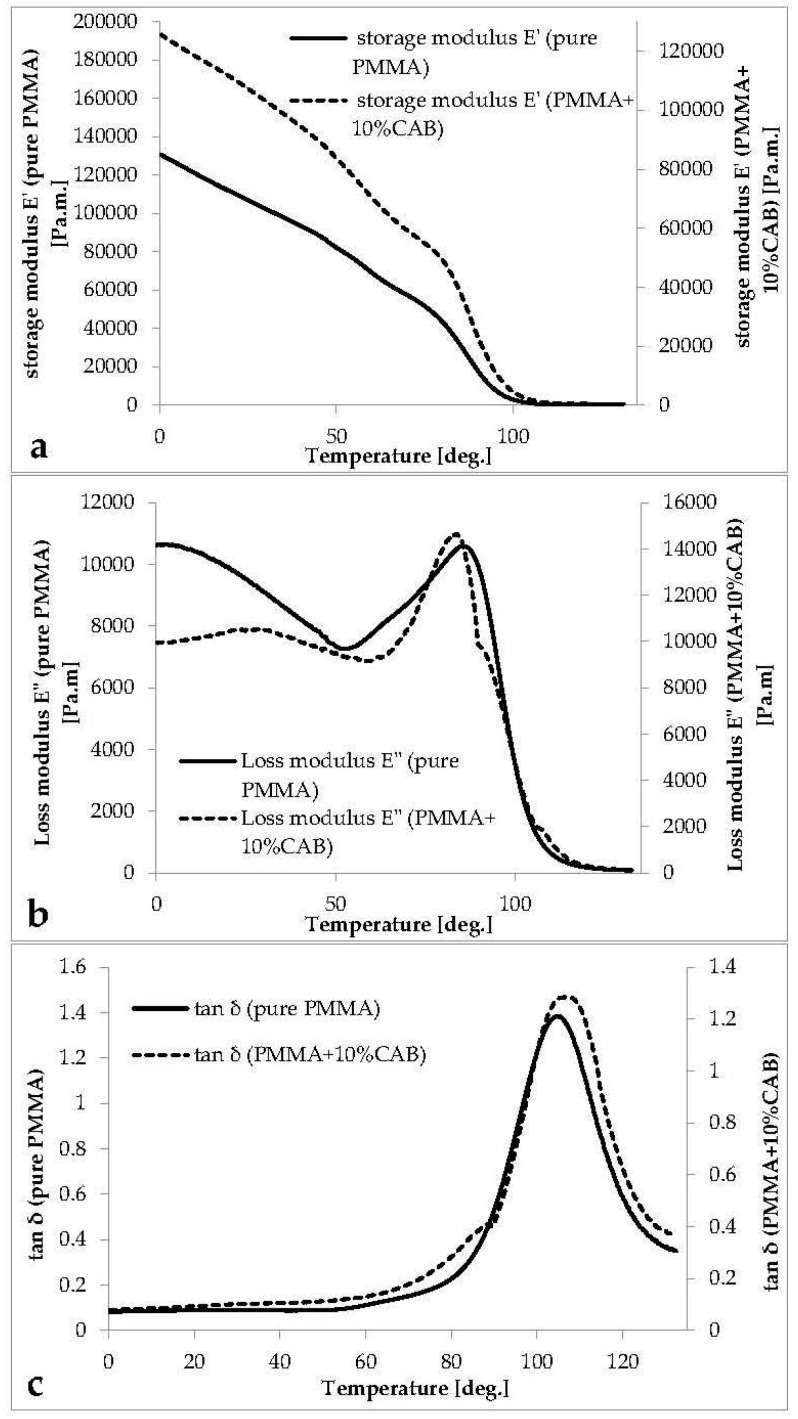
Pure PMMA and PMMA/10%CAB trace for (**a**) storage modulus E'; (**b**) loss modulus E"; and (**c**) the loss factor (tan δ).

The curves of the storage modulus E' (the ability of the material to store potential energy), loss modulus E" (energy dissipation in the form of heat upon deformation), and phase angle tan δ (the mechanical damping or internal friction in a viscoelastic system) *vs.* temperature for pure PMMA, and PMMA/10%CAB blends are shown in (Figure 4).

The storage modulus curve for PMMA and PMMA/10%CAB (Figure 4a) shows three main regions (glassy, transition, and rubbery). In the glassy region, there was a sharp decline in the E' curve down to the transition region without any notable signs of the three deformation phases in this region. The E' curve at 80° centered a transition region (primary transition area/α-relaxation) with a steep slope. α-relaxation is attributed to main chain motion (the glass-rubber transition) [33,34]. At 115 °C deformation was highly viscoelastic (rubbery plateau region), indicating main chain mobility (large scale chain mobility). Flow (melt) was evident at 130 °C; in this area the bonds become a slipping case [32].

There was a slight increase in modulus after CAB addition to PMMA, which led to an increase in the material’s ability to store potential energy (stiffness). Moreover, it should be noted that in going from pure PMMA to PMMA/10%CAB, the hydroxyl groups’ mole fraction increases. Similarly, due to the hydrogen bonding between the hydroxyl groups, the steric limitation to polymer chain motion is probable, which may slow the relaxation frequency [34]. The two-step reduction in the loss modulus curve (as shown in Figure 4b) after adding 10% CAB is characteristic of an immiscible blended system [35]. The mechanical loss factor (tan δ) curve (Figure 4c) shows the same divergence at 89 °C near the *T*_g_ area. The peak at the lower temperature for PMMA/10%CAB was a signature of a secondary relaxation or β-relaxation process [33]. The tan δ curve peak at 105 °C for PMMA and PMMA/10%CAB represents the glass transition temperature (*T*_g_) for pure and blended (atactic) PMMA [36].

### 3.5. Thermogravimetric Analysis

**Figure 5:**
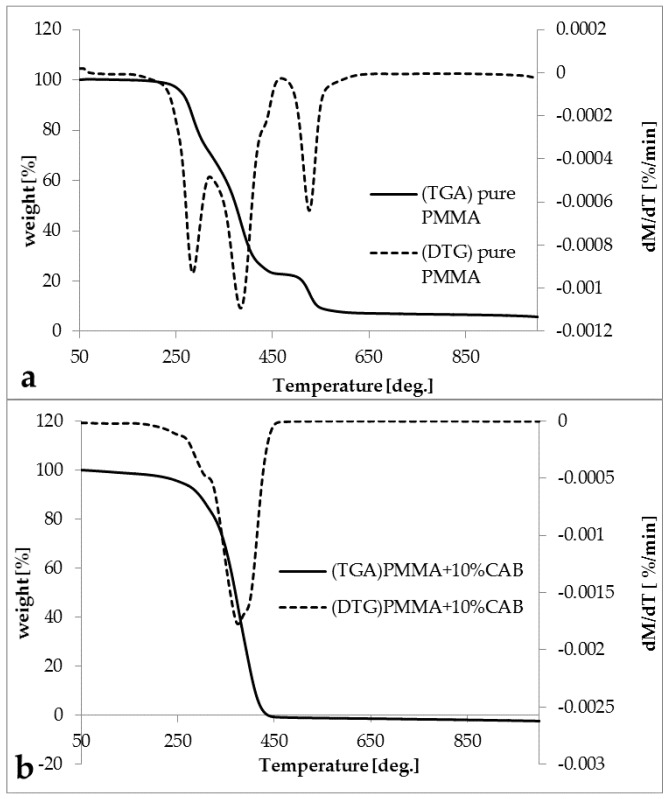
TGA- trace for (**a**) pure PMMA; and (**b**) PMMA/10%CAB.

The thermogravimetric analysis (TGA) and derivative thermogravimetry (DTG) diagrams for pure PMMA and the PMMA/10%CAB blend are shown in (Figure 5a,b). All of the DTG peaks represent the degradation point for each stage. It was observed that, for pure PMMA, there were three degradation steps at 280, 384, and 525 °C, respectively, indicating that PMMA was radically polymerized [37]. The first stage with a total mass loss ≈ 27.45% was due to head-to-head linkage cleavage (H–H linkage), the second stage with a total mass loss ≈ 47.82% was due to the degradation of unsaturated vinylidene ends, thus cleavage from the chain-end and the third stage (the residual polymer) with a total mass loss ≈ 15.57% was equivalent to the decomposition of the main chain of PMMA [37,38,39,40]. PMMA/10%CAB blend showed a single degradation stage at 370 °C because of the organic constituents’ degradation [41].

Multi-stage processes, such as those observed in pure PMMA, may cause differences in apparent activation energy values over the transformation range; however, unequivocal interpretation of such changes is not easy. Thus, 10%CAB inhibits activation energy values of the blend [42]. The break in each thermogram indicates the onset of the decomposition process involving a rapid loss in weight [43].

## 4. Conclusions

Environmentally friendly transparent PMMA/CAB blends were successfully prepared by melt-blending in a twin screw extruder. The polymer blends were found to be immiscible (PMMA/CAB) with different levels of mixing. The properties of the blends were not only a function of the blend composition but also clearly depended on the degree of dispersion of CAB in PMMA, as well as the phase interaction between the components of the blend. The incorporation of CAB into PMMA reduced the absorption of ultraviolet rays at the damage threshold for both polymers, while the amorphous state for PMMA remained the same after adding CAB. The best blend sample contained 10% *w*/*w* CAB in the PMMA/CAB blend, demonstrating high transparency and low ultraviolet light absorption. The results also showed clear improvement in some properties, such as an increase in modulus, loss factor, stiffness, thermal stability, and glass transition temperature for PMMA/10%CAB blend.

## References

[1] Guo H.F., Packirisamy S., Mani R.S., Aronson C.L., Gvozdic N.V., Meier D.J. (1998). Compatibilizing effects of block copolymers in low-density polyethylene/polystyrene blends. Polymer.

[2] Xing C., Wang H., Hu Q., Xu F., Cao X., You J., Li Y. (2013). Mechanical and thermal properties of eco-friendly poly(propylene carbonate)/cellulose acetate butyrate blends. Carbohydr. Polym..

[3] Chen Y.-H., Chen L.-L., Shang N.-C. (2009). Photocatalytic degradation of dimethyl phthalate in an aqueous solution with Pt-doped TiO_2_-coated magnetic PMMA microspheres. J. Hazard. Mater..

[4] Osawa Z., Fukuda Y. (1991). Photo-degradation of blends of polycarbonate and poly(methyl methacrylate). Polym. Degrad. Stab..

[5] Ibrahim N., Khalil H., Eid B. (2015). A cleaner production of ultra-violet shielding wool prints. J. Clean. Prod..

[6] Tayyar A.E., Alan G. (2015). Outdoor usage performances of woven fabrics dyed with self-cleaning dyes. J. Text. Inst..

[7] Varganici C.-D., Rosu L., Mocanu O.M., Rosu D. (2015). Influence of poly(vinyl alcohol) on cellulose photochemical stability in cryogels during UV irradiation. J. Photochem. Photobiol. A Chem..

[8] Chen B., Zhong L., Gu L. (2010). Thermal properties and chemical changes in blend melt spinning of cellulose acetate butyrate and a novel cationic dyeable copolyester. J. Appl. Polym. Sci..

[9] Ball F.M., Coney C.H. (1959). Sulfonic Acid Catalyzed Cellulose Acetate Butyrate Urea-Formaldehyde Coating Composition for Paper and Process of Preparation. U.S. Patent.

[10] Coney C.H. (1965). Cellulose Acetate Butyrate Emulsion Coating. U.S. Patent.

[11] Rauner F.J., Rosati I.F., Robertson E.M. (1963). Dry Processed Photothermographic Printing Plate and Process. US Patent.

[12] Agruss M.S. (1964). Light Sensitive Triphenylmethane Leucocyanide Compositions. U.S. Patent.

[13] Dever J., Banks B., de Groh K., Miller S., Kutz M. (2005). Degradation of spacecraft materials. Handbook of Environmental Degradation of Materials.

[14] Shultz A.R. (1961). Degradation of polymethyl methacrylate by ultraviolet light. J. Phys. Chem..

[15] Fox R.B., Isaacs L.G., Stokes S. (1963). Photolytic degradation of poly(methyl methacrylate). J. Polym. Sci. Part A Gen. Pap..

[16] Caykara T., Güven O. (1999). UV degradation of poly(methyl methacrylate) and its vinyltriethoxysilane containing copolymers. Polym. Degrad. Stab..

[17] Wochnowski C., Eldin M.S., Metev S. (2005). UV-laser-assisted degradation of poly(methyl methacrylate). Polym. Degrad. Stab..

[18] Charlesby A., Thomas D. (1962). A comparison of the effects of ultra-violet and gamma radiation in polymethylmethacrylate. R. Soc..

[19] Michelson J., Werner L., Ollerton A., Leishman L., Bodnar Z. (2012). Light scattering and light transmittance in intraocular lenses explanted because of optic opacification. J. Cataract Refract. Surg..

[20] Abouelezz M., Waters P.F. (1978). Studies on the Photodegradation of Poly(Methyl Methacrylate).

[21] Fekete E., Pukanszky B. (2005). Effect of molecular interactions on the miscibility and structure of polymer blends. Eur. Polym. J..

[22] Shih J.S., Musa O.M. (2014). Ultraviolet-Absorbing Compounds. US Patent.

[23] Bath P., Romberger A., Brown P. (1986). A comparison of Nd: YAG laser damage thresholds for PMMA and silicone intraocular lenses. Investig. Ophthalmol. Vis. Sci..

[24] Silverstein R.M., Webster F.X., Kiemle D., Bryce D.L., David L.B. (2014). Spectrometric Identification of Organic Compounds.

[25] Song M., Park M.S., Kim J.K., Cho B., Kim K.H., Sung H.J., Ahn S. (2005). Water-soluble binder with high flexural modulus for powder injection molding. J. Mater. Sci..

[26] Sarı A., Alkan C., Karaipekli A., Uzun O. (2010). Poly(ethylene glycol)/poly(methyl methacrylate) blends as novel form-stable phase-change materials for thermal energy storage. J. Appl. Polym. Sci..

[27] Selvakumar M., Krishna Bhat D. (2008). Miscibility of poly(methylmethacrelate) and cellulose acetate butyrate blends in dimethyl formamide. Indian J. Chem. Technol..

[28] Bhat D.K., Kumar M.S. (2006). Biodegradability of PMMA blends with some cellulose derivatives. J. Polym. Environ..

[29] Elizalde-Pena E., Flores-Ramirez N., Luna-Barcenas G., Vásquez-García S.R., Arámbula-Villa G., García-Gaitán B., Rutiaga-Quiñones J.G., González-Hernández J. (2007). Synthesis and characterization of chitosan-*g*-glycidyl methacrylate with methyl methacrylate. Eur. Polym. J..

[30] Silvestre C., Cimmino S., Martuscelli E., Karasz F.E., MacKnight W.J. (1987). Poly(ethylene oxide)/poly(methyl methacrylate) blends: Influence of tacticity of poly(methyl methacrylate) on blend structure and miscibility. Polymer.

[31] Mathur A., Bhardwaj I., Mathur A., Jyoti M. (2003). Testing and Evaluation of Plastics.

[32] Menard K.P. (2008). Dynamic Mechanical Analysis: A Practical Introduction.

[33] Dixit M., Gupta S., Mathur V., Rathore K.S., Sharma K., Saxena N.S. (2009). Study of glass transition temperature of PMMA and CdS-PMMA composite. Chalcogenide Lett..

[34] Merenga A.S., Katana G. (2010). Dynamic mechanical analysis of PMMA-cellulose blends. Int. J. Polym. Mater..

[35] Ward I.M., Sweeney J. (2012). Mechanical Properties of Solid Polymers.

[36] Teng H., Koike K., Zhou D., Satoh Z., Koike Y., Okamoto Y. (2009). High glass transition temperatures of poly(methyl methacrylate) prepared by free radical initiators. J. Polym. Sci. Part A Polym. Chem..

[37] Kashiwagi T., Inaba A., Brown J.E., Hatada K., Kitayama T., Masuda E. (1986). Effects of weak linkages on the thermal and oxidative degradation of poly(methyl methacrylates). Macromolecules.

[38] Kashiwagi T., Morgan A.B., Antonucci J.M., Van Landingham M.R., Harris R.H., Awad W.H., Shields J.R. (2003). Thermal and flammability properties of a silica-poly(methylmethacrylate) nanocomposite. J. Appl. Polym. Sci..

[39] Morgan A.B., Antonucci J.M., Van Landingham M.R., Harris R.H., Awad W.H., Shields J.R. (2000). Thermal and flammability properties of a silica-PMMA nanocomposite. Polym. Mater. Sci. Eng. Wash..

[40] Raouf R.M., Wahab Z.A., Ibrahim N.A., Talib Z.A., Chieng B.W. (2016). Miscible transparent polymethylmethacrylate/cellulose acetate propionate blend: optical, morphological, and thermomechanical properties. BioResources.

[41] Wojciechowska P., Foltynowicz Z. (2009). Synthesis of organic-inorganic hybrids based on cellulose acetate butyrate. Polimery.

[42] Kandare E., Deng H., Wang D., Hossenlopp W.M. (2006). Thermal stability and degradation kinetics of poly(methyl methacrylate)/layered copper hydroxy methacrylate composites. Polym. Adv. Technol..

[43] Tosh B. (2011). Thermal analysis of cellulose esters prepared from different molecular weight fractions of high a-cellulose pulp. Indian J. Chem. Technol..

